# Evaluation of Apical and Molecular Effects of Algae *Pseudokirchneriella subcapitata* to Cerium Oxide Nanoparticles

**DOI:** 10.3390/toxics11030283

**Published:** 2023-03-19

**Authors:** Ntombikayise Mahaye, Ndeke Musee

**Affiliations:** Emerging Contaminants Ecological and Risk Assessment (ECERA) Research Group, Department of Chemical Engineering, University of Pretoria, Pretoria 0028, South Africa; mahaye.ntombi@gmail.com

**Keywords:** *Pseudokirchneriella subcapitata*, cerium oxide nanoparticles, DNA stability, RAPD-PCR, growth effect, chlorophyll *a* content

## Abstract

Cerium oxide engineered nanoparticles (nCeO_2_) are widely used in various applications and are, also, increasingly being detected in different environmental matrixes. However, their impacts on the aquatic environment remain poorly quantified. Hence, there is a need to investigate their effects on non-target aquatic organisms. Here, we evaluated the cytotoxic and genotoxic effects of <25 nm uncoated-nCeO_2_ on algae *Pseudokirchneriella subcapitata*. Apical (growth and chlorophyll *a* (Chl *a*) content) and genotoxic effects were investigated at 62.5–1000 µg/L after 72 and 168 h. Results demonstrated that nCeO_2_ induced significant growth inhibition after 72 h and promotion post 96–168 h. Conversely, nCeO_2_ induced enhanced Chl *a* content post 72 h, but no significant changes were observed between nCeO_2_–exposed and control samples after 168 h. Hence, the results indicate *P. subcapitata* photosynthetic system recovery ability to nCeO_2_ effects under chronic-exposure conditions. RAPD-PCR profiles showed the appearance and/or disappearance of normal bands relative to controls; indicative of DNA damage and/or DNA mutation. Unlike cell recovery observed post 96 h, DNA damage persisted over 168 h. Thus, sub-lethal nCeO_2_-induced toxicological effects may pose a more serious threat to algae than at present anticipated.

## 1. Introduction

Cerium oxide engineered nanoparticles (nCeO_2_) are a class of emerging contaminants with unique properties when compared to their bulk counterpart. These properties include redox activity, scavenging of free radicals, and inhibition of biofilm formation [1]. As a result, nCeO_2_ find widespread applications, e.g., in sunscreens as UV absorbent [2], fuel additives [3], catalysis [4,5], biomedicine [6], and nano-pharmacy [7]. Estimates indicate that nCeO_2_ global production to be 100 and 1000 tons/year [8] and may have increased to 7500–10,000 tons/year [9,10]. Since nCeO_2_ is widely used as an additive for diesel fuels, for instance, it is, therefore, among likely engineered nanoparticles (ENPs) of ecological concern in the natural environment [11]. In addition, nCeO_2_ has been quantified in various environmental matrixes using modelling (e.g., 0.1 µg/L in surface waters [12] and experimental (0.4–5.2 ng/L in surface waters [13]) approaches. However, the environmental implications of nCeO_2_ remain poorly understood. Yet, it is among the top ten priority ENPs identified both for the evaluation of human health and environmental safety effects [14].

Following the release of nCeO_2_ into the ecosystems, they may accumulate in the aquatic systems linked to their transformation processes including aggregation, sedimentation, and low degradation rate. This, in turn, increases their uptake, accumulation, and bio-magnification in the food chain [15] and inevitable interactions with different classes of aquatic organisms [16]. Therefore, understanding the impact of nCeO_2_ on aquatic biota especially organisms that represent the base of the trophic chain is of ecological significance.

Algae are unicellular organisms and primary producers that play a vital role in the structure and functioning of ecosystems and are susceptible test species to environmental pollutants [17]. In fact, to date, several studies have documented the deleterious effects of nCeO_2_ on algae [16,18,19,20,21]. For example, following exposure of freshwater algae *Pseudokirchneriella subcapitata* over 72 h to poly acrylic acid (PAA) coated-nCeO_2_ (4–10 nm; spherical shaped) exposure at concentrations of 15–200 µg/L, significant oxidative stress response was observed, and EC_50_ of 24 µg/L was reported owing to the ENPs’ dispersion and bioavailability [18]. Most effect studies of nCeO_2_ on algae were conducted using apical endpoints (e.g., growth effects), with only a handful at the sub-lethal level [22,23] and at low environmentally relevant concentrations (ng/L to µg/L).

Remarkably, at sub-lethal exposure concentrations, the ENPs effects at the morphological level generally are masked but are apparent at the molecular level [24,25,26,27]. For instance, Taylor et al. [26] assessed the molecular and phenotypic toxic effects of 4–5 nm polyvinylpyrrolidone (PVP)-coated nCeO_2_ (0.5–80,000 µg/L) on freshwater algae *Chlamydomonas reinhardtii* using transcriptomic and metabolomic techniques. Results demonstrated the internalization of mono-dispersed PVP-nCeO_2_ by intracellular vesicles, but no growth inhibition was observed irrespective of exposure concentration. Additionally, molecular perturbations (e.g., down-regulation of photosynthesis) were observed only at very high concentrations (>10,000 µg/L). Based on their results, the authors recommended the assessment of longer-term exposure and consequences of internalization within the aquatic food chain, bioavailability, and potential toxicity of nCeO_2_ to primary consumers [26]. Importantly, ENPs may undergo dissolution upon exposure to aqueous media. These metal ions in suspensions of metal-based ENPs play an important role in determining toxicity of ENPs [28]. For example, following exposure of algae to nCeO_2_, the Ce concentration in the medium decreased [20]. These results showed that nCeO_2_ and Ce ions might adsorb on the algal cell surface or enter algal cells. The intracellular available Ce content in the 50 mg/L treatment was significantly higher than the control, which might do harm to algal cells. The authors of [29] reported that Ce^3+^ of 0.5~10 mg/L could inhibit the growth of *Anabaena flosaquae*. Meanwhile, Ce could enter the cell of *Arabidopsis thaliana* and destroy the ultrastructure of cells [30].

Conversely, Angel et al. [31] reported IC_50_ of *P. subcapitata* of dissolved Ce (0.63 mg/L) to be much higher than the measured solubility. Thus, they considered that the dissolved Ce could hardly cause the observed toxicity. In a study by Wu et al. [20], the intracellular Ce content of 50 mg/L nCeO_2_ treatment was the highest among all the treatments, which might be responsible for the growth inhibition and toxicity effects of nCeO_2_. The authors concluded that, however, it is difficult to determine whether the adsorption of nCeO_2_ or the intracellular Ce contribute more to the toxicity of nCeO_2_ [20]_._

Overall, documented studies have demonstrated the importance of molecular studies towards robust risk assessment of ENPs on the aquatic biota. This is why, in recent years, ecotoxicological studies have shifted from apical to molecular endpoints including for ENPs [22]. This is because changes at the molecular level have been demonstrated to induce deleterious long-term ecological implications [32] not observable at an organismal level. Among the molecular effect assays, includes genotoxicity-based methods. For example, random amplified polymorphic deoxyribonucleic acid by Polymerase Chain Reaction (RAPD-PCR) analysis has been widely applied to assess the genotoxicity of ENPs to aquatic biota, e.g., algae [27], aquatic invertebrates [33], and fish [34,35]. The key advantage of RAPD-PCR analysis is its ability to screen changes in DNA profiles and evaluate genomic stability. For instance, Mahaye and colleagues recently applied RAPD-PCR and the apurinic/apyrimidinic (AP) sites techniques to evaluate the genotoxic effects of differently coated gold nanoparticles (nAu) on algae *P. subcapitata* over 168 h. The genotoxicity results demonstrated significant toxicity of nAu on algae including on samples where undesirable effects were undetectable from the apical endpoints (e.g., growth effect and chlorophyll *a* (Chl *a*) content) [27].

At present, there are a lack of genotoxicity data pertaining to the interactions between nCeO_2_ and algae unlike in the case of crustaceans and fish, especially for nTiO_2_ and nAg as the most studied organisms and ENPs, respectively [22]. To address this knowledge gap, herein we investigated the impact of nCeO_2_ on freshwater microalgae *P. subcapitata* at low exposure concentrations in the µg/L range as their use increases; thus, they are likely to be found in the actual environment. The study specific objectives were to assess the effects of nCeO_2_ (at concentrations of 62.5–1000 µg/L) on *P. subcapitata*: (i) at apical endpoints including growth effect and Chl *a* content and (ii) on DNA integrity using RAPD-PCR analysis in 10% Blue Green algae-11 (BG-11) medium after 72 and 168 h. The toxicological effects observed from the molecular and apical endpoints assessments were compared or linked to gain better understanding on the effects of nCeO_2_ on algae under chronic exposure conditions.

## 2. Materials and Methods

### 2.1. Characterization of nCeO_2_

Uncoated nCeO_2_ in dispersion (<25 nm particle size, 10 wt.% in H_2_O) were purchased from Sigma Aldrich (Johannesburg, South Africa). Size and morphology were characterized as previously described by Mahaye [36]. Hydrodynamic diameter (HDD) and zeta (ζ) potential of nCeO_2_ in de-ionized water (DI water) (15 MΩ/cm) and 10% Blue Green algae-11 media (herein referred to as BG-11 media) [37] were measured using dynamic light scattering (DLS) (Malvern Zetasizer Nano ZS, Malvern, UK). Measurements for the ζ-potential and HDD were taken at 0, 2, 6, 24, 48, and 72 h in triplicate. The HDD and ζ-potential measurements in DI water and BG-11 media were only done at 1000 µg/L nCeO_2._ This is because nCeO_2_ at concentrations <1000 µg/L were below the detection limit using the Zetasizer.

### 2.2. Preparation of Exposure Media and Concentrations

Algal experiments were conducted in BG-11 media (media preparation and composition details are listed in Section SI-1 and Table S1, respectively, in the supporting information) following Direct Estimation of Ecological Effects Potential (DEEEP) toxicity testing protocols [37]. The media was stored at 4 °C under dark conditions before use. The nCeO_2_ exposure concentrations of 62.5, 125, 250, 500, and 1000 µg/L were prepared in BG-11 media in triplicate and ultra-sonicated for 30 min before carrying out the exposure experiments.

### 2.3. Test Organisms

The Algaltoxkit F^TM^ kit (MicroBioTests Inc., Gent, Belgium) was purchased from ToxSolutions (Johannesburg, South Africa). Algaltoxkit F contains all the materials needed to perform a 72 h growth inhibition tests with the freshwater microalgae *P. subcapitata* (former names: *Selenastrum capricornutum* and *Raphidocelis subcapitata*). This algal toxicity test was carried strictly in adherence to ISO Standard 8692 and OECD Guideline 201 protocols. The de-immobilization of *P. subcapitata* from the beads was performed in accordance with the manufacturer’s instructions. In this study, stock cultures were incubated under controlled conditions (temperature: 25 ± 1 °C; light intensity: 6000 Lux; 12:12 h light: dark cycle and shaken continuously at 100 rpm) for 5–7 d to obtain exponentially growing *P. subcapitata*.

### 2.4. Cytotoxic Effects of nCeO_2_ on P. subcapitata

Preparation of the algal test was performed as outlined in Mahaye et al. [27]. Reference tests aimed to ascertain the sensitivity of algae growth are described in Section SI-2. Inoculum was prepared by harvesting exponentially growing *P. subcapitata* cells. Cells from a 5–7 d old stock culture prepared in Section 2.3 were transferred as 1 mL volume into Eppendorf tubes and centrifuged at 10,000 rpm for 10 min. The supernatant was decanted, and the algal cells were re-suspended in 0.1 mL phosphate-buffered saline (PBS). The centrifugation and decanting steps were repeated twice. The volume of stock culture required and the cell density of algal inoculum required per experiment in test and control wells were calculated using the following expression:

(1) $$Volume\;\left(\mathrm{mL}\right)=\frac{no.\;of\;flasks\;used\times \frac{vol}{flask}\times 200000\;cells/mL\;}{Cell\;density\;\left(\mathrm{cells}/\mathrm{mL}\right)\;in\;the\;stock\;culture}$$

where vol/flask is the volume of test solution per flask and cells/mL is the cell density in the inoculum given by the following expression [38]:

(2) $$\mathrm{Cells}/\mathrm{mL}=e^{\frac{ln\lambda_{684}+16.439}{1.0219}}$$

where λ_684_ is the optical density (OD) at 684 nm.

In the final step, algal cells were re-suspended and mixed well in 10% BG-11 media and the cell density in the inoculum was measured before the experiment was initiated. For each test, 200,000 cells/mL sample was required. Tests were carried out in 2 mL volumes in 24-well microplates with 1.8 mL test sample (or de-ionised water for the control), with 0.2 mL of the inoculum and algal medium. Thereafter, it was incubated at the same conditions as the stock culture for 168 h. *P. subcapitata* exposures to nCeO_2_ were conducted following the standard algal test of 72 [37], or 96 h [39] with slight modifications. First, the exposure time was increased from 96 to 168 h to gain insights on likely effects under chronic conditions. The standard US EPA flask test method [39] yielded inadequate biomass for genotoxicity analysis as it requires only 10,000 cells/mL as initial inoculum. Thus, to generate sufficient biomass for genotoxicity analysis, we used the DEEEP toxicity testing protocol [37]. This protocol requires an inoculum of 200,000 cells/mL, which, in turn, generated adequate biomass for DNA damage analysis [27].

For negative controls, exposures for algae were done without nCeO_2_. All experiments were done in triplicate. Exponentially growing *P. subcapitata* were exposed to five concentrations of nCeO_2_ (62.5, 125, 250, 500 and 1000 µg/L) for 168 h, in a 24-well microplate system, under defined conditions outlined in Section 2.3. Exposure concentrations were selected based on the detected or predicted environmental concentrations from the previous studies. For example, nCeO_2_ concentrations of 0.3–230 µg/L [40] and 0.04–0.27 μg/L [41] were reported in freshwater. The ENPs’ concentrations in freshwater are predicted to reach six-fold higher by the year 2050 [42]. Thus, in this study, the selected exposure concentrations cover both current and plausible future predicted concentrations of nCeO_2_ in freshwater systems.

After the experiments were initiated, the cell density (in the form of optical density) was measured at 684 nm every 24 h for 168 h using a microplate reader (FLUOstar Omega BMG Labtech, Ortenberg, Germany). Briefly, the wavelength of 684 nm used here was adopted from Rodrigues et al. [38] and has been successfully used on ENPs-exposed *P. subcapitata* studies [27,43,44]. After 72 and 168 h exposure periods, Chl *a* content was determined following a protocol by Harris [45]. Briefly, 1 mL of the control and exposed algal cells were centrifuged for 10 min at 13,000 rpm, and the pellet was washed using DI water. The algal cells pellet was suspended in 95% ethanol, vortexed for 2 min, kept at 4 °C for 30 min and centrifuged at 13,000 rpm for 2 min. The supernatant was analysed for Chl *a* content using a UV-Vis spectrophotometer (HACH, Loveland, CO, USA) at wavelengths of 665 and 649 nm. The content of Chl *a* was then calculated using the expression:

(3) $$\mathrm{Chl}\;a=13.70A_{665}-5.76A_{649}$$

where *A*_665_ and *A*_649_ are the OD values (*n* =3) at wavelengths of 665 nm and 649 nm, respectively.

### 2.5. DNA Damage and Estimation of Genomic Template Stability

DNA isolation, visualization, and amplification were done as described in Mahaye et al. [27], and details are set out in Section SI-3. Briefly, exponentially growing *P. subcapitata* were exposed to three nCeO_2_ concentrations at 62.5, 250, and 1000 µg/L as described in Section 2.3. RAPD-PCR data analysis was performed by comparing the PCR product profiles for nCeO_2-_treated sample with the control samples. The genomic template stability percentage (GTS%) was calculated using the following Equation [46]:

(4) $$\mathrm{GTS}=\left(1-\frac{a}{n}\right)\times 100$$

where *a* is the average number of RAPD polymorphic bands detected in ENPs-treated samples and *n* is the total bands in the controls. Polymorphisms in RAPD profiles include deletion of a normal band and induction of a new band in comparison to the control RAPD profiles. GTS percentage of nCeO_2_ -treated samples was calculated and changes of genomic stability were expressed as a percentage of controls set at 100%.

### 2.6. Data Analysis

All measurements were performed in triplicate, and the results were expressed as mean ± standard deviation (SD). Statistical analysis was performed using GraphPad Prism Software version 9.3.0 (GraphPad Software, San Diego, CA, USA). One-way analysis of variance (ANOVA) followed by Dunnett’s post hoc test was used to evaluate statistical differences between nCeO_2_-exposed samples and the controls. Differences between samples were considered statistically significant when *p* ≤ 0.05.

## 3. Results and Discussion

### 3.1. Characterization of nCeO_2_

**Figure 1:**
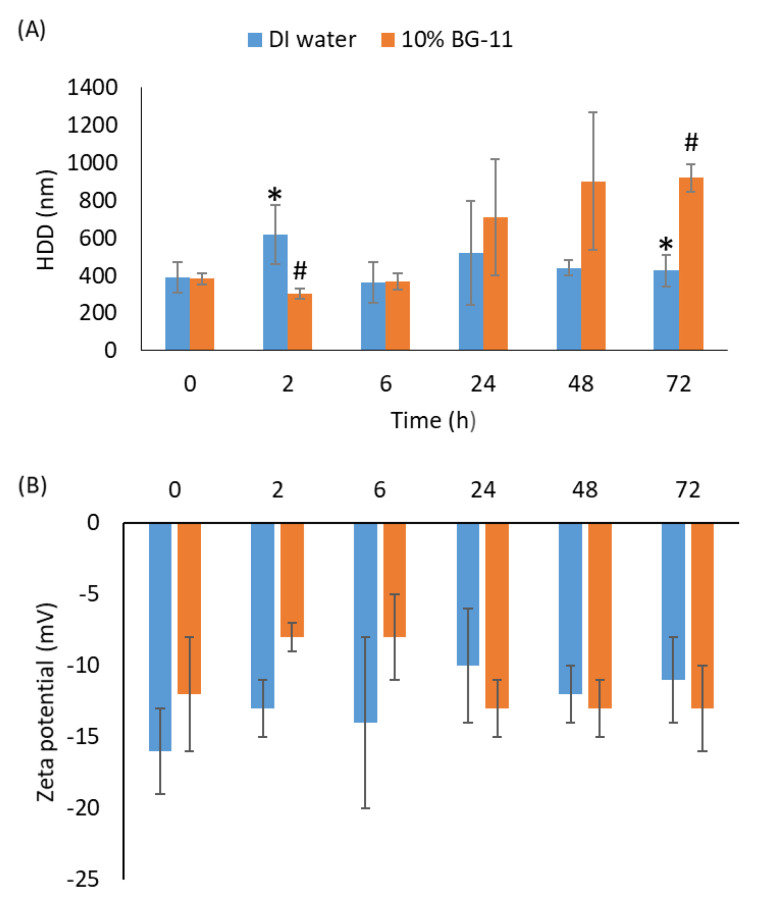
The (**A**) HDD and (**B**) ζ potential at 1000 µg/L of nCeO_2_ in DI water and BG−11 media measured using DLS over 72 h. Data are presented as mean ± standard deviation (SD). Different symbols denote significant differences (*p* < 0.05) between DI water and BG-11 media per time period analysed using Two-way ANOVA with Tukey’s multiple comparisons test.

nCeO_2_ had non-uniform triangular, tetrahedral, and hexagonal shapes (Figure S1a), with diameters of 15–50 nm due to the asymmetry of the morphology. Although most nCeO_2_ were <25 nm, larger and compact crystalline structures were also observed (Figure S1b). Figure S1c depicts the particle size distribution of nCeO_2_ at 1000 µg/L in BG-11 media measured using DLS. The presence of agglomerates >25 nm showed that the primary particle sizes could not be attained even after ultrasonication, as previously documented in other works [47,48,49,50]. nCeO_2_ aggregated immediately following introduction into both DI water and BG-11 media (Figure 1A). After 24–72 h exposure, aggregation was higher in BG-11 media compared to DI water (Figure 1A). The higher aggregation was likely due to high ionic strength of BG-11 media relative to DI water as previously documented [51,52].

In an earlier work, uncoated nCeO_2_ sized 28 nm was observed to form aggregates of 200–300 nm in ultrapure water [49]. Here, the observed aggregation in BG-11 media show a good agreement with the behaviour of uncoated-nCeO_2_ as documented in other algal ecotoxicity media, e.g., synthetic freshwater algal media [31,53], OECD TG 201 [54] and Dutch Standard (DS) medium [55]. For example, 20 nm uncoated-nCeO_2_ immediately agglomerated to 218 nm in DS medium, which is ten-fold higher than the primary size [55]. Negative ζ-potential values for 1000 μg/L nCeO_2_ were observed in both media types over 72 h and at a narrow range of −8 to −16 mV (Figure 1B). These low ζ-potential values indicate nCeO_2_ instability and are consistent with the rapid agglomeration observed in both DI water and BG-11 media (Figure 1A). This is because ζ-potential values should be ±30 mV to stabilize ENPs suspensions [56,57]. 

### 3.2. Effect of nCeO_2_ on Algal Growth

**Figure 2:**
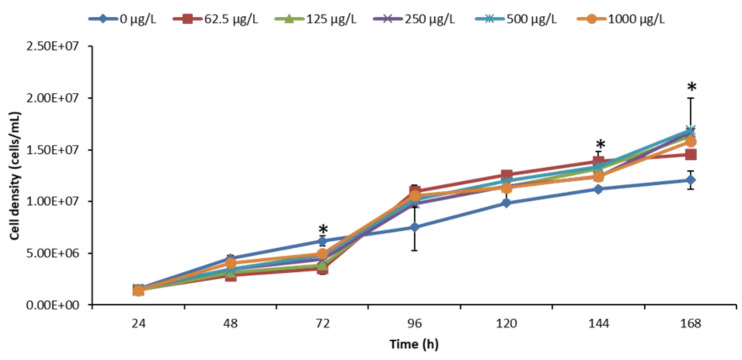
Cell density of *P. subcapitata* at different exposure concentrations of nCeO_2_. Results were reported as mean ± standard deviation where *n* = 3. The asterisk denotes significant differences (*p* < 0.05) between nCeO_2_-treated and control samples.

A positive control was performed using potassium dichromate (K_2_Cr_2_O_7_) as a reference toxicant, and results are presented in Figure S2. Results in Figure 2 demonstrates the growth effect of nCeO_2_ on algae over 168 h. Remarkably, nCeO_2_ had no significant effect on algal growth following exposure after 24 and 48 h but induced significant growth inhibition after 96 h relative to the controls. Growth promotion was, however, observed after 96 h and significantly higher after 144 and 168 h, compared to the controls. Any modification of algae growth may, subsequently, affect higher trophic levels [58]. For instance, they may lead to altered species composition and habitat structure [59] and, as a result, compromise ecological integrity. Among ecological functioning, aspects that may be adversely affected include the extinction of sensitive algal species and macrophytes, or higher growth may outcompete other biological life forms with consequent undesirable perturbations on the food chain and nutrient recycling, among others.

Similar to our findings, Dedman et al. [60] investigated growth effects of *Prochlorococcus* sp. MED4 by <25 nm nCeO_2_ over 72 h (1–100 µg/L) and extended exposure time of 240 h at environmentally relevant (1–100 µg/L) and supra-environmental (1–100 mg/L) concentrations. Results indicated significant reduction of *Prochlorococcus* cell density (up to 68.8%) at 100 µg/L nCeO_2_ after 72 h. The lowest tested concentrations of 1 and 10 μg/L induced no observable effect on *Prochlorococcus* growth irrespective of exposure time (72 and 240 h). However, 1 μg/L induced about 38.8% increase in cell density relative to the control in nutrient-enriched media after 240 h. Exposure to supra-environmental nCeO_2_ concentrations (i.e., 100 mg/L) yielded a significant decline in cell density of up to 95.7 and 82.7%, respectively, in natural oligotrophic seawater and nutrient-enriched media. The observed cell decline was attributed to the extensive aggregation behaviour of nCeO_2_ upon entry into natural seawater and hetero-aggregation with algae [60]. In addition, direct contact of nCeO_2_ with algae was reported previously to be responsible for toxicity and to cause membrane damage of *P. subcapitata* [53]. Further, an increase in intracellular reactive oxygen species (ROS) was observed in algae [19]. Intracellular ROS plays a role in the inhibition of photosynthesis and can indicate oxidative damage [61].

Previous studies have demonstrated the absence of uptake of uncoated and agglomerated nCeO_2_ in algae [31,61]. The lack of ENPs uptake was linked to the formation of agglomerates that exceeded the pore sizes (ranges between 5 and 20 nm) of the algal cell wall [62], which, in turn, impeded plausible uptake by algae. Here, the observed agglomerates (up to 918 ± 74 nm) exceeded the algal cell wall pore sizes and, therefore, uptake was reasonably unlikely. Thus, growth inhibition observed was possibly due to the entrapment of algal cells by ENPs agglomerates. As a result, this may have reduced the light and nutrients’ availability to the entrapped algal cells with concomitant growth inhibition. Previously, algal growth inhibition was observed to result from physical removal due to co-aggregation and co-sedimentation with nCeO_2_, as opposed to the toxicological and cell death effect [60].

nCeO_2_ effects on algae have been observed to be concentration- and exposure duration-dependent [60,63,64]. For example, growth inhibition of the algae *Microcystis aeruginosa* following exposure to < 25 nm nCeO_2_ (1, 10 and 50 mg/L) in BG-11 media over 72 h was observed to be exposure-duration dependent [64]. No significant differences were observed between the controls and nCeO_2-_treated samples after 24 h. However, after 48 h at concentrations of 1 and 10 mg/L nCeO_2_, results indicated algal growth promotion, but 50 mg/L induced significant growth inhibition of *M. aeruginosa* [64]. After 72 h, no algal growth was observed at 1 mg/L, increased significantly at 10 mg/L, but a significant inhibition was apparent at 50 mg/L nCeO_2_ [64]. Deng and colleagues reported similar results, where they observed induction of growth promotion on marine diatom *Phaeodactylum tricornutum* at low nCeO_2_ concentrations of ≤5 mg/L, whereas growth inhibition at ≥10 mg/L was documented [63].

Herein, the observed recovery of algal population under extended exposure conditions may be attributed to a decrease in nCeO_2_ concentrations bioavailable for algae (Figure S3) as ENPs formed aggregates (Figure 1A and Figure S1) and underwent sedimentation in BG-11 media over time. Furthermore, recovery of the populations was attributed to the algae defence mechanisms in response to ENPs exposure. For example, algae employ a variety of defence mechanisms including activation of the antioxidative defence system to eliminate reactive oxygen species (ROS) [65,66], excretion of biomolecules to form a protective layer [67], and intracellular processes to decrease the cellular content of ENPs [68]. Thus, the findings herein and others demonstrates that the likely environmental risk of nCeO_2_ on algae appear to be low at the morphological level even under extended exposure conditions.

### 3.3. Effect of nCeO_2_ on Chl a Content

**Figure 3:**
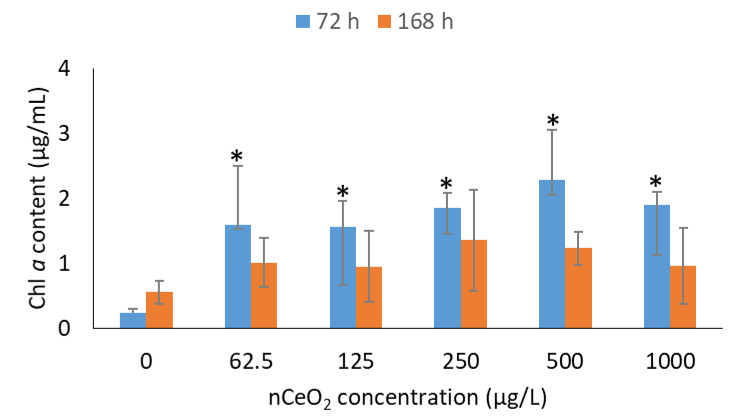
Chl *a* content of *P. subcapitata* at different nCeO_2_ exposure concentrations. Results are reported as mean ± standard deviation where *n* = 3 and asterisk denotes significant differences (*p* ≤ 0.05) between exposed and non-exposed samples after 72 h.

Photosynthesis is a key process in algae and quantified as Chl *a* content–an efficient indicator for physiological health status of algal cells [69,70]. Figure 3 demonstrates Chl *a* content of *P. subcapitata* for nCeO_2_- and non-exposed samples after 72 and 168 h. Contrary to the algal growth inhibition observed up to 72 h (Figure 2), findings in Figure 3 demonstrate that nCeO_2_ enhanced Chl *a* content (*p* < 0.05) compared to the controls over the same period, but remarkably independent of the exposure concentration. In an earlier work, increase in Chl *a* content relative to controls were observed on *C. reinhardtii* following exposure to 4 nm-sized uncoated-nCeO_2_ at 0.1–50 mg/L [16]. The observed increase in Chl *a* was associated with an interruption of the electron transport at the acceptor side of photosystem PSII [71,72]. Furthermore, other metal oxide ENPs, e.g., nZnO and nTiO_2_, were observed to enhance algal growth and Chl *a* content in *Picochlorum* sp. [73] and *P. subcapitata* [74,75,76]. The basis for Chl *a* content promotion was plausibly due to the conversion of other forms of pigments (e.g., Chl *b* content) into Chl *a* content as a response to ROS following exposure to ENPs [77]. Gui et al. [78] reported significant increases in Chl content after plant exposure to 10, 50, and 100 mg/kg nCeO_2_ after 40 d. On day 50, only 50 and 100 mg/kg nCeO_2_ concentrations increased the Chl content. Similar to our findings after 168 h, at the harvest stage, all of nCeO_2_ treatments had no more significant difference [78]. After nCeO_2_ (200 mg/L) exposure for 1 w, the Chl *a* and Chl *b* contents of rice seedlings did not show any significant changes relative to the control [79].

After 168 h, no significant changes in Chl *a* content were observed between nCeO_2_–exposed and control samples. These findings indicated that the photosynthetic system of *P. subcapitata* can tolerate the presence of nCeO_2_ under chronic exposure conditions. In contrast to growth promotion post 96 to 168 h, a reduction in Chl *a* content was observed after 168 h compared to 72 h. Similarly, the findings of Zhao et al. [64] showed that 10 mg/L nCeO_2_ promoted algal growth, but it was also accompanied by a slight inhibition of photosynthetic yield. In addition, exposure of *P. subcapitata* to <50 nm uncoated-nCeO_2_ at 0.01–100 mg/L for 72 h showed a dual response, firstly, with 20–50% stimulation in Chl *a* content at lower concentration range of 0.01–1 mg/L and, secondly, a significant inhibition was observed at higher concentrations of 10–100 mg/L [19]. The primary cause of the observed photosynthetic inhibition was due to excessive levels of ROS formation, which, in turn, induced oxidative damage as evidenced by lipid peroxidation data [19,69]. Furthermore, ENPs bound onto algal membranes were observed to induce a shading effect or membrane damage and, in turn, inhibit the photosynthesis process [80,81,82]. To date, physical restraints and oxidative stress were reported to be mechanisms responsible for ENPs toxicity to algae [83]. The entrapment of algal cells by large ENP aggregates not only reduces light available for photosynthesis, but also prevents uptake of nutrients [70]. Among available ENP-toxicity mechanisms, a large number of studies indicated oxidative stress as the dominant toxicity mechanism of ENPs to algae [84]. For instance, Chen et al. [85] conducted a meta-analysis study, and the results showed that the level of ROS significantly increased by 90% in the presence of ENPs, indicating the accumulation of excess ROS in algal cells which ultimately caused oxidative stress. Additionally, ENP-induced ROS accumulation was not significantly influenced by ENP surface modification (*p* = 0.103) but was strongly influenced by the ENP type (*p* = 0.044), ENP dose (*p* = 0.001), and algae species (*p* < 0.001). Findings of the current study point to the need to consider long-term exposure conditions, as the results of nCeO_2_ on algae appear to be exposure time dependent.

### 3.4. DNA Damage and Estimation of Genomic Template Stability

**Figure 4:**
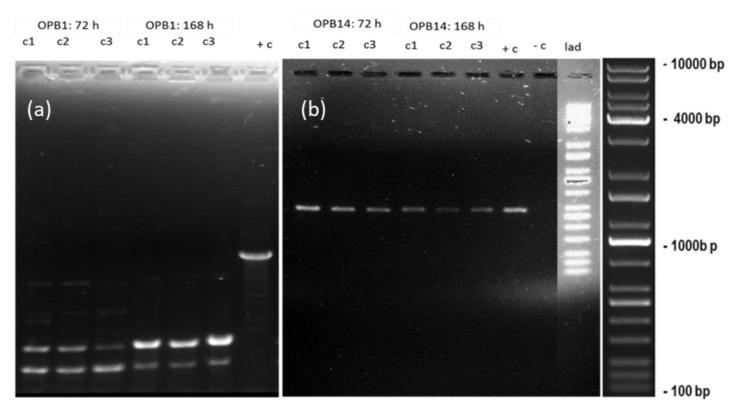
RAPD-PCR profiles generated using (**a**) OPB1 primer and (**b**) OPB14 primer after 72 and 168 h. Abbreviations: c1—62.5 µg/L; c2—250 µg/L; c3—1000 µg/L; +c—untreated control; −c—negative control (no DNA; and lad − DNA ladder.

Cytotoxicity study (growth effect and Chl *a* content) results did not differ as a function of nCeO_2_ exposure concentration compared to the control (Figure 2 and Figure 3). Thus, genotoxicity studies using RAPD-PCR method were conducted at 62.5, 250, and 1000 µg/L nCeO_2_ representing the lowest, median, and highest concentrations, correspondingly. The results in Figure 4 show the RAPD-PCR profiles of isolated genomic DNA from nCeO_2_-treated and untreated samples. These profiles were also used to analyse GST% (Equation (2)). A negative control (no DNA) was included to ascertain whether any band observed was attributable to DNA amplification.

RAPD-PCR profiles for nCeO_2_ treated algae using OPB1 primer were markedly different from those of the controls (Figure 4a). Modifications in DNA were in the form of appearance of two new clear bands at ±200–500 bp and disappearance of a normal clear band observed at ±900 bp in the controls (Figure 4a). Specifically, we observed various size ranges for nCeO_2_ treated samples compared to the controls. Notably, the DNA strands in the form of clear bands for nCeO_2_ treated samples were shorter (±200–500 bp) compared to the controls (±900 bp), indicating that the DNA of the control samples was more intact compared to one from nCeO_2_ treated algae. The observed DNA modifications were neither concentration nor time dependent, indicating that nCeO_2_-induced 72 h-DNA damage persisted over 168 h. The observed modifications of RAPD-PCR profiles were likely due to one or a combination of variant events, e.g., DNA adducts, DNA breakage and mutation (e.g., point mutations and large rearrangements) [46,86]. The OPB14 primer produced similar RAPD profiles for controls and nCeO_2_-treated algal DNA irrespective of exposure concentration and time (Figure 4b), with a GTS of 100%. Similarly, RAPD profile analysis after exposure of *Pseudomonas putida* to aluminium oxide ENPs (nAl_2_O_3_) showed no difference to the control, pointing to the induction of the DNA repair mechanisms [87]. Furthermore, the findings demonstrated primer-dependent genotoxicity. Previously, exposure of *P. putida* bacteria to <50 nm nAl_2_O_3_ using four primers, e.g., OPA2, OPA10, OPA9, and OPA18 also showed primer-dependent DNA damage [87]. Results obtained using primer OPA2, demonstrated the most significant mutagenic action of nAl_2_O_3_, whereas the OPA10 and OPA18 primers RAPD band profiles showed the least mutagenic effect with small variations between ENPs-treated samples and control.

Similarly, using the OPB1 primer, Mahaye et al. [27] observed DNA bands characterized by various size ranges compared to the controls following exposure of *P. subcapitata* to citrate- and branched-polyethyleneimine-nAu at 62.5–1000 µg/L for 168 h. The OPB14 primer produced similar RAPD-PCR profiles, irrespective of nAu coating type and exposure duration or concentration. The results of Mahaye et al. [27] indicated that DNA stability decreased after 72 h and increased after 168 h. Thus, they were indicative of likely DNA damage recovery over a long-term exposure period. However, herein, findings for nCeO_2_ demonstrated persistent DNA damage under extended exposure conditions. This is critical, as genotoxic effects may be, subsequently, transmitted to future generations with deleterious implications such as a compromised defence towards pests or an inability to adopt adverse environmental conditions.

In turn, this may affect survival and reproduction of algae, thus, compromising ecological balance as algae are food source for higher organisms in the food web. For example, transfer of metal oxide ENPs from algae to daphnia [88] or algae to fish [89] have been reported. In addition, the findings imply that high agglomeration of nCeO_2_ in BG-11 medium does not reduce their reactivity and genotoxicity. These findings indicate that DNA damage on algae is ENPs type dependent. Previous findings have demonstrated irreparable DNA damage where affected cells can trigger cell death by activation of apoptosis to eliminate potentially damaged cells [90]. Conversely, herein, findings from apical endpoints after 168 h (Figure 2 and Figure 3) plausibly indicate cell recovery under chronic conditions compared to 72 h. Metagenomic analysis results have demonstrated that microbial communities can protect themselves and recover their functions through keystone taxa, development of resistance, and resilience and functional redundancy [91]. The findings emphasize the importance of including genotoxicity methods in the risk assessment of ENPs on algae. Furthermore, current findings contribute to the limited body of knowledge on the effects of nCeO_2_ on algae at low exposure concentrations (µg/L) and long-term exposure conditions, especially using the multi-maker approach that coupled genotoxicity biomarkers with apical endpoints to aid gain complete picture on the effect of these emerging contaminants.

## 4. Conclusions

nCeO_2_ at 62.5–1000 µg/L exposure concentrations induced significant algal growth inhibition after 72 h, but growth promotion post 96–168 h, irrespective of exposure concentration. After 72 h, nCeO_2_ enhanced Chl *a* content compared to the controls at all tested concentrations. However, after 168 h, no significant changes in Chl *a* content were observed between non-exposed and nCeO_2_–exposed samples (*p* > 0.05). The findings demonstrated that the high agglomeration of smaller-sized nCeO_2_ do not reduce their reactivity nor hinder their toxicological effects. Furthermore, growth results demonstrated that algal cells could recover under long-term exposure conditions (post 96 h). Assessment of DNA damage using RAPD-PCR showed DNA bands modifications in the form of appearance of new bands and/or disappearance of normal bands compared to the controls. The observed modifications of RAPD-PCR profiles point to likely DNA adducts, DNA breakage, and mutation (point mutations and large rearrangements). In contrast to cell recovery observed after 96 h, DNA damage persisted over 168 h.

Overall, the study provided evidence that exposure duration plays a vital role on the cytotoxic and genotoxic response of *P. subcapitata* to nCeO_2_. To fully understand the mechanism of ENPs toxicity in algae, we recommend further studies at different endpoints at the molecular (e.g., chromosomal abnormalities, nucleus damage, DNA strand breaks, gene expression) and biochemical (e.g., catalase (CAT), glutathione *S*-transferase (GST), superoxide dismutase (SOD), etc.) levels. Furthermore, studies should be carried out at low environmentally relevant ENPs concentrations using a more realistic exposure medium (e.g., river water) and under chronic exposure conditions to fully understand the long-term impact of nCeO_2_ on non-target aquatic organisms.

## Data Availability

Not available.

## Supplementary Materials

The following supporting information can be downloaded at: https://www.mdpi.com/article/10.3390/toxics11030283/s1, Table S1: The composition of 10% BG-11 medium; Figure S1: Size characterization of nCeO2 (a) TEM images [36], (b) size distribution; Figure S2: Algal growth of *P. subcapitata* at different concentrations of K_2_Cr_2_O_7_: Figure S3: in situ nCeO_2_ concentration (particles/mL) characterization examined using Nanoparticle Tracking Analysis [92].
