# Supplementary material for: Evaluation of Apical and Molecular Effects of Algae Pseudokirchneriella subcapitata to Cerium Oxide Nanoparticles
Source: Toxics. 2023 Mar 19;11(3):283. doi: 10.3390/toxics11030283 (PMC10058573; doi:10.3390/toxics11030283)
Supplement: Supplementary file 1 [file toxics-11-00283-s001.zip › toxics-2217800-supplementary.pdf]

Evaluation of apical and molecular effects of algae *Pseudokirchneriella subcapitata* to cerium oxide nanoparticles

Ntombikayise Mahaye, Ndeke Musee

Emerging Contaminants Ecological and Risk Assessment (ECERA) Research Group, Department of Chemical Engineering, University of Pretoria, Private Bag X20, Hatfield, Pretoria 0028, South Africa
* Correspondence: ndeke.musee@up.ac.za or museen2012@gmail.com

S1-1 Exposure medium preparation

Chemicals for the 10% BG-11 media: ($CuSO_4.5(H_2O)$, $ZnSO_4.7(H_2O)$, Co $(NO_3)_2.6H_2O$, $C_6H_8O_7$, $NaHCO_3$, $NaNO_3$, $CaCl_2{\cdot}2(H_2O)$, $MgSO_4{\cdot}7(H_2O)$, K_2HPO_4, Fe (NH_3)-citrate, $Na_2EDTA{\cdot}2(H_2O)$, H_3BO_3, $MnCl_2{\cdot}4(H_2O)$, and $Na_2MoO_4{\cdot}2(H_2O)$)) were purchased from Sigma Aldrich (Johannesburg, South Africa) and were used without further purification. The 10% BG-11 media chemicals were dissolved in 1 L DI water (15 MΩ.cm) in sterile autoclaved pre-cleaned glassware, the pH was adjusted to 7-7.5, and the media was autoclaved before used.

Table S1: The composition of 10% BG-11 medium

	Macronutrients	Volume (mL) per 1 L
A	$CaCl_2.2H_2O$	1
B	$NaNO_3$	10
C	K_2HPO_4	1
D	$MgSO_4{\cdot}7(H_2O)$	1
E	$NaHCO_3$	1
	Micronutrients	
F	H_3BO_3<br>$MnCl_2{\cdot}4(H_2O)$<br>$ZnSO_4{\cdot}7H_2O$<br>$CuSO_4{\cdot}5H_2O$<br>$Co(NO_3)_2.\ 6H_2O$<br>$NaMoO_4.2H_2O$<br>Citric acid	1
G	Na_2-EDTA.$2H_2O$	1
H	$Fe(NH_3)$-citrate	1

SI-2 Algal reference test with potassium dichromate

To assess the test sensitivity, the positive control was performed using potassium dichromate ($K_2Cr_2O_7$) as a reference toxicant. Exponentially growing *P. subcapitata* were exposed to varied $K_2Cr_2O_7$ concentrations (0.32 -3.2 mg/L) over 72 h. Cell density readings were measured at OD_{684} every 24 h, and the 50% effective concentration (EC_{50}) was determined. Herein, a concentration dependent growth inhibition of *P. subcapitata* due to $K_2Cr_2O_7$ compared to control was observed over 72 h as shown in Figure S2. The EC_{50} value of 0.31 mg/L was determined and considered to be in good agreement with 0.43 mg/L reported in the test kit.

SI-3 Optimization of PCR conditions for RAPD assay

Four 10-base pairs RAPD primers, namely, OPA9 (5'-GGGTAACGCC -3'), OPB10 (5'-CTGCTGGGGAC-3'), OPB1 (5'-GTTTCGCTCC-3'), and OPB14 (5'-TCCGCTCTGG-3'), were initially tested for RAPD-PCR. This is because these primers have been previously applied in genotoxicity studies, including algal studies (Atienzar et al. 2002; Nyati et al. 2013; Mahaye et al. 2021). However, herein only two primers showed stable results as well as greater

reproducibility and were selected for this study, viz.: OPB1 and OPB14. Primers were purchased from Inqaba Biotechnical Industries (Pty) Ltd (South Africa).

Amplification was performed in 25 µL reaction volumes consisting of 0.5 µL primers, 10 µL DI water, 12.5 µL GoTaq® G2 Hot Start Green Master Mix (GoTaq® G2 Hot Start Polymerase, dNTPs, $MgCl_2$, and reaction buffers: Promega, USA), and 2 µL genomic DNA using a Polymerase Chain Reaction (PCR) thermocycler (T100™ Thermal Cycler). For the negative control, genomic DNA was not added and DIW was used to make up the 25 µL volume. The PCR steps consisted of DNA denaturation for 5 min at 95 °C, followed by 30 cycles of 40 s at 95 °C, 40s at 56 °C, and 2 min at 72 °C, then a final extension for 10 min at 72 °C. The amplified DNA was separated at 90 mV for 1 h and visualised using a UV transilluminator. Following these PCR steps, no bands were obtained in 1% and 1.5% ethidium bromide-stained agarose gel dissolved in 1 X TAE buffer (Tris-acetate-EDTA buffer, pH 7.5).

A gradient PCR was then used to find optimum annealing temperatures for each primer. A temperature range between 35 and 50° C was selected based on primer melting temperatures as provided by the supplier (39.5 °C for OPB1 and 43.6 °C for OPB14). The annealing temperatures tested for each primer were in the range of 35-50 °C. The RAPD-PCR protocol consisted of the following steps: a 35-cycle warming step at 95 °C for 5 min, DNA denaturation at 95°C for 1 min, annealing (30 –50 °C) for 1 min, extension 74 °C for 1 min, and final extension at 74 °C for 10 min. The amplified product was gel electrophoresed at 80 mV for 2 h and visualised using a UV transilluminator. No DNA bands were observed at any of the tested temperatures. Following the same procedure, the study was repeated with bovine serum albumin (BSA) to enhance binding between the DNA and primer at an annealing temperature range of 35–50 °C (50.0, 48.7, 46.8, 44.3, 40.8, 37.8, 35.9, 35.5 and 35.0 °C). A comparison of results generated with and without BSA, showed the former had clear bands, and, hence, BSA was used in this study as DNA-primer binding enhancer. The annealing temperatures (46.8 °C for OPB1 and 44.3 °C for OPB14) with more visible bands for each primer were selected for the experiments.

SI-3.1 DNA isolation and visualization

After exposing *P. subcapitata* to $nCeO_2$ over 72 and 168 h, algal DNA was extracted using the MasterPure™ DNA purification kit (Epicentre, USA) following the manufacturer's instructions. Importantly, three minor modifications were made (Mahaye et al. 2021). These

entailed: first, sample incubation was increased from 15 min to 30 min at 65 °C to improve cell lysis. Second, 1 µL was increased to 3 µL RNase cocktail to increase the efficiency of cell lysis. Finally, an additional 1 h incubation step at -20 °C was done after the addition of isopropanol to improve DNA precipitation. DNA precipitation and purification were done in accordance with the prescribed manufacturer's protocol. DNA purity (OD_{260}/OD_{280}) and concentration (ng/µL) were determined using a Nanodrop 2000 Spectrophotometer (Thermo Scientific, US). The ratio of $OD_{260}/OD_{280} \geq 1.7$ denoted contaminant-free DNA, and < 1.7 signified the presence of contaminants (Barbas et al., 2007). Three-µL of the extracted DNA was separated using 1.5% ethidium-bromide-stained agarose gel dissolved in 1 X TAE buffer (Tris-acetate-EDTA buffer, pH 7.5) at 90 mV for 45 min. Thereafter, DNA visualization was done using a UV transilluminator (Bio-Rad, USA).

SI-3.2 DNA amplification

Amplification was performed in 25 µL reaction volumes consisting of 1 µL primers, 6 µL DI water, 2.5 µL bovine serum albumin (BSA), 12.5 µL ready mix, and 3 µL genomic DNA using a PCR thermocycler (T100™ Thermal Cycler, Bio-Rad, Singapore). For the negative control, no genomic DNA was added. Further, amplification using the RAPD primers was carried out under the following conditions: warming step at 94 °C for 5 min, 40 cycles of denaturation at 94 °C for 45 s, annealing (46.8 °C for OPB1 and 44.3 °C for OPB14) for 30 s, extension 68 °C for 1 min, and a final extension at 68°C for 10 min. The amplified products were kept at 4 °C until gel electrophoresis analysis was performed. Bands were separated on a 1.5% ethidium bromide-stained agarose gel at 80 mV for 2 h and then visualised using a UV transilluminator (Bio-Rad, USA). A 10 kb universal DNA Ladder (Kapa Biosystems, South Africa) was used as the molecular weight standard. RAPD data analysis was performed by comparing the PCR product profiles for treated and control samples. The GTS% was calculated using Eq. (2) (Atienzar et al. 2002).

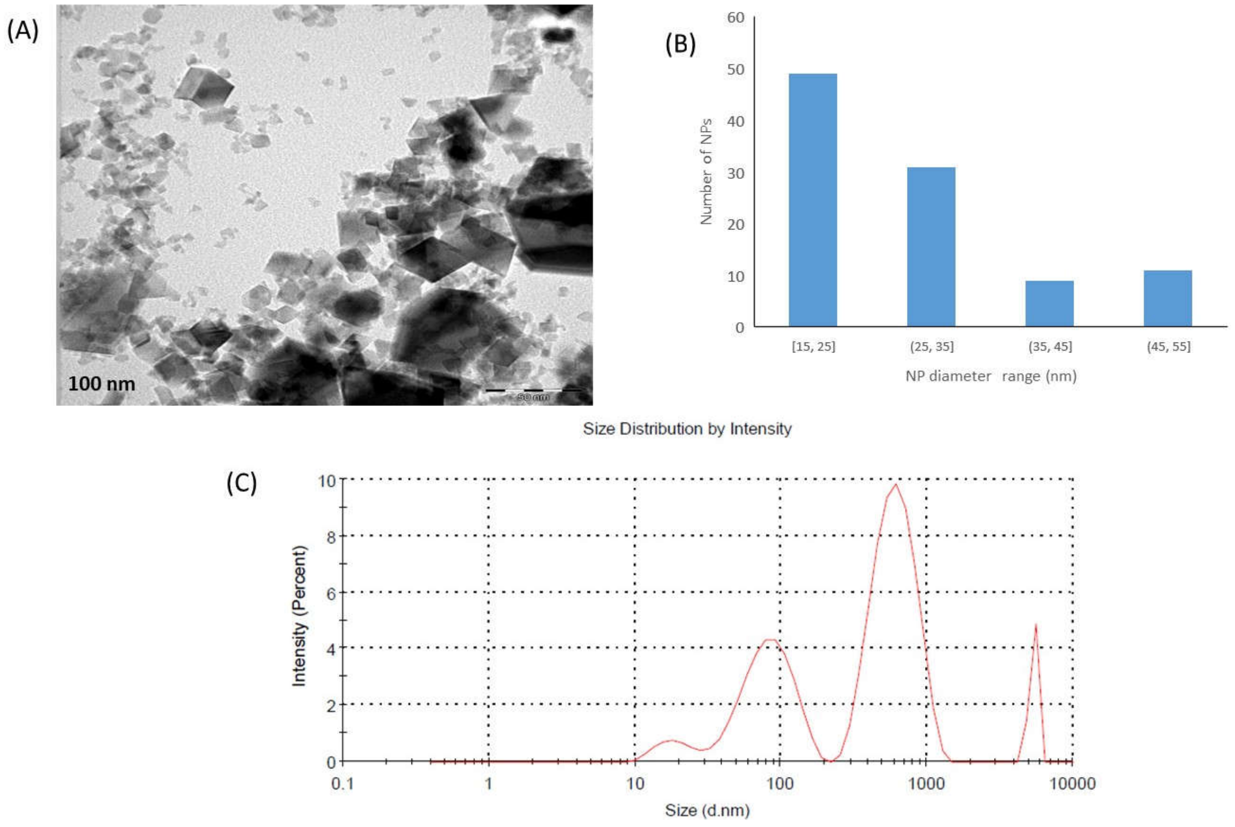

Figure S1: Size characterization of $nCeO_2$ (**A**) TEM images (Mahaye, 2019), (**B**) size distribution (n =100) ("n = 100" refers to the total number of individual $nCeO_2$ measured from TEM images to give the reported mean sizes), and (**C**) particle size distribution of $nCeO_2$ at 1000 μg/L in 10% BG-11 media measured using the Dynamic Light Scattering technique at 0 h.

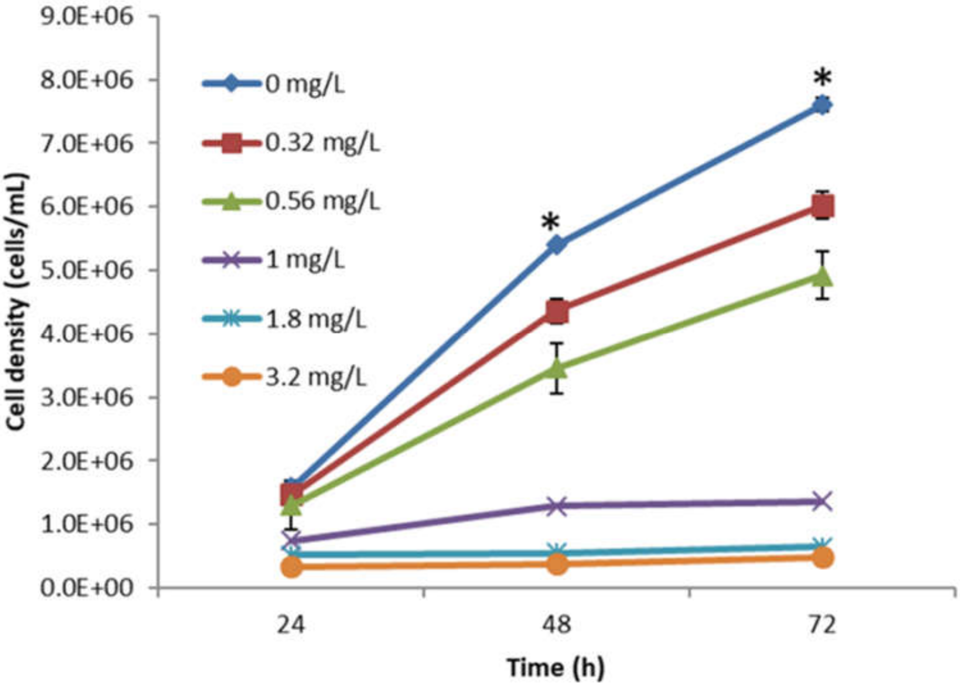

Figure S2: Algal growth of *P. subcapitata* at different concentrations of $K_2Cr_2O_7$. Bars denote standard deviation where $n = 3$, and * denotes significant differences ($p < 0.05$) between the control and treated samples. (Mahaye et al. 2021)

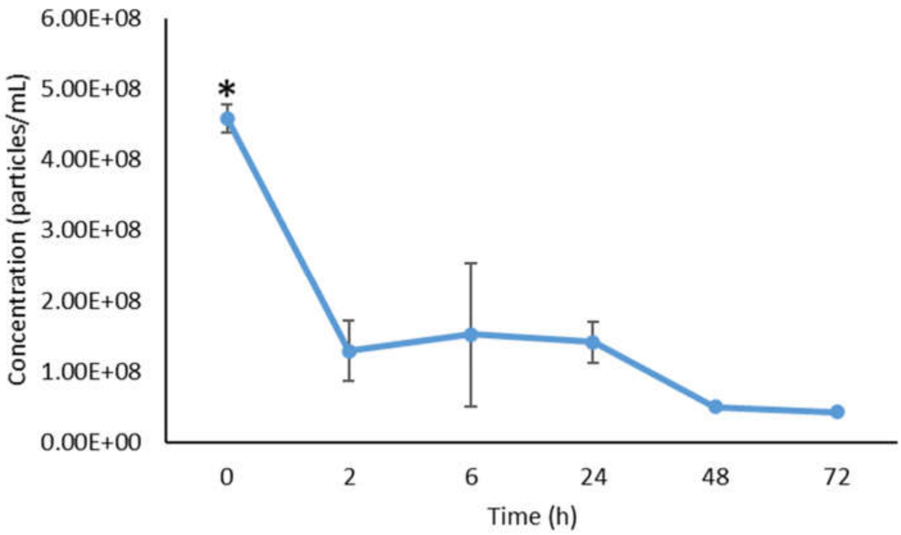

Figure S3: *in situ* $nCeO_2$ concentration (particles/mL) characterization examined using Nanoparticle Tracking Analysis (NanoSight NS500, NTA 3.0 Software, Amesbury, UK). Results are represented as mean (n =3), and bars denote standard deviation. The * denotes

statistical differences ($p < 0.05$) between concentrations over time analysed using one-way analysis of variance (ANOVA), followed by Dunnett's post hoc test.

References

Atienzar, F.A.; Venier, P.; Jha, A.N.; Depledge, M.H. Evaluation of the Random Amplified Polymorphic DNA (RAPD) Assay for the Detection of DNA Damage and Mutations. *Mutation Research/Genetic Toxicology and Environmental Mutagenesis* **2002**, *521*, 151–163, doi:10.1016/S1383-5718(02)00216-4.

Nyati, S.; Werth, S.; Honegger, R. Genetic Diversity of Sterile Cultured Trebouxia Photobionts Associated with the Lichen-Forming Fungus Xanthoria Parietina Visualized with RAPD-PCR Fingerprinting Techniques. *The Lichenologist* **2013**, *45*, 825–840.

Mahaye, N.; Leareng, S.K.; Musee, N. Cytotoxicity and Genotoxicity of Coated-Gold Nanoparticles on Freshwater Algae Pseudokirchneriella Subcapitata. *Aquatic Toxicology* **2021**, *236*, 105865, doi:10.1016/j.aquatox.2021.105865.

Mahaye, N. Stability of Gold and Cerium Oxide Nanoparticles in Aqueous Environments, and Their Effects on *Pseudokirchneriella Subcapitata* and Salvinia Minima. PhD, University of Pretoria: Pretoria, 2019.
